# Clinical Utility of NGS-Based Diagnosis in Primary Ciliary Dyskinesia: Experience from a Brazilian Pediatric Cohort at a Reference Center for Rare Diseases

**DOI:** 10.3390/genes17070767

**Published:** 2026-06-30

**Authors:** Patrícia F. Barreto M. Costa, Danielle de Freitas F. M. Fins, Isabelle de Oliveira Moraes, Tania Wrobel Folescu, Renata Wrobel Folescu Cohen, Natana Chaves Rabelo, Leticia Azevedo Barreto, Julia Vieira da Cunha Moreira, Bianca Barbosa Abdala, Mariana Naccarato Teixeira Lopes Andrade, Juan Llerena, Dafne Horovitz, Maria Eduarda Gomes, Sayonara Gonzalez

**Affiliations:** 1 Setor de Pneumologia, Departamento de Pediatria, Instituto Fernandes Figueira (IFF/FIOCRUZ), Serviço de Referência para Doenças Raras, Ministério da Saúde, Rio de Janeiro 22250-020, RJ, Brazil; pneumopatriciabarreto@gmail.com (P.F.B.M.C.); tania.folescu@gmail.com (T.W.F.); renatawfc@gmail.com (R.W.F.C.); leticiaab2003@gmail.com (L.A.B.); vieirajulia.cm@gmail.com (J.V.d.C.M.); mari_ntl@yahoo.com.br (M.N.T.L.A.); 2 Laboratório de Biologia Molecular/Medicina Genômica, Centro de Genética Médica, Instituto Fernandes Figueira (IFF/FIOCRUZ), Serviço de Referência para Doenças Raras, Ministério da Saúde, Rio de Janeiro 22250-020, RJ, Brazil; danielle.fins@fiocruz.br (D.d.F.F.M.F.); isabelle.moraes@fiocruz.br (I.d.O.M.); natana.rabelo@fiocruz.br (N.C.R.); bianca.abdala@fiocruz.br (B.B.A.); 3 Instituto Nacional de Doenças Raras (InRaras), Hospital das Clínicas de Porto Alegre, Porto Alegre 90035-903, RS, Brazil; 4 Unidade de Genética Clínica, Centro de Genética Médica, Instituto Fernandes Figueira (IFF/FIOCRUZ), Serviço de Referência para Doenças Raras, Ministério da Saúde, Rio de Janeiro 22250-020, RJ, Brazil; juan.llerena@fiocruz.br (J.L.J.); dafne.horovitz@fiocruz.br (D.H.); 5 Centro Universitário Arthur Sá Earp Neto/Faculdade de Medicina de Petrópolis (UNIFASE), Petrópolis 25680-120, RJ, Brazil

**Keywords:** primary ciliary dyskinesia, exome sequencing (ES), next-generation sequencing (NGS), bronchiectasis, ciliopathies, rare genetic diseases

## Abstract

**Background**: Primary ciliary dyskinesia (PCD) is a rare and genetically heterogeneous disorder that remains underdiagnosed in low- and middle-income countries, largely due to limited access to specialized diagnostic tests. Genetic analysis has become an essential component of PCD diagnosis, particularly where functional and ultrastructural evaluations are unavailable. **Methods**: We conducted an investigational study including children and adolescents with clinical suspicion of PCD followed at a Brazilian tertiary center. Clinical characterization included detailed phenotyping and calculation of the PICADAR score. Molecular investigation was performed using exome sequencing as a frontline diagnostic approach. **Results**: Among 27 individuals evaluated, 10 (37%) received a confirmed molecular diagnosis of PCD. An additional 6 (22%) individuals had inconclusive molecular findings, mainly due to variants of uncertain significance (VUS), and were classified as likely PCD based on combined clinical and molecular evidence. Higher PICADAR scores were more frequently observed among individuals with confirmed or likely molecular diagnosis, with 9 of 10 confirmed cases presenting a score above 5. Beyond PCD-associated findings, exome sequencing also enabled the identification of clinically relevant additional diagnoses, including cystic fibrosis, *FGFR3*-related hypochondroplasia, and ACMG-reportable secondary finding involving *BRCA2*. Some unresolved cases may also reflect inherent technical limitations of exome sequencing, including restricted sensitivity for copy-number variants, suboptimal coverage of highly homologous or GC-rich regions, and limited detection of deep intronic and other variants. Additional factors include challenges in variant interpretation and incomplete knowledge of disease-associated genes. **Conclusions**: Frontline exome sequencing is a valuable diagnostic tool for PCD, particularly when integrated with robust clinical phenotyping. Clinical scoring systems such as PICADAR may help prioritize individuals for genetic testing and optimize diagnostic yield in resource-limited settings.

## 1. Introduction

Primary ciliary dyskinesia (PCD) is a rare and clinically and genetically heterogeneous ciliopathy caused by impaired structure and function of motile cilia, resulting in defective mucociliary clearance and multisystem involvement [1,2]. The estimated global prevalence is approximately 1 in 7500 individuals, although this figure varies substantially across populations and is likely underestimated due to diagnostic challenges and limited access to specialized testing [3].

PCD exhibits a broad phenotypic spectrum, encompassing neonatal respiratory distress in term infants, chronic upper and lower airway disease with recurrent respiratory infections, progressive bronchiectasis, and chronic rhinosinusitis [2,4]. Approximately 50% of affected individuals present with laterality defects, including *situs inversus totalis* and *situs ambiguus*, reflecting impaired nodal ciliary function during embryonic left–right patterning [5,6]. Additional manifestations may occur depending on the ciliated tissues involved and include subfertility in both sexes due to dysfunctional motile cilia in the reproductive tract, congenital heart disease and, more rarely, hydrocephalus related to ependymal ciliary dysfunction [4]. This multisystem involvement leads to phenotypic overlap with other genetic and respiratory disorders, such as cystic fibrosis (CF), primary immunodeficiencies, non-CF bronchiectasis, and laterality disorders, which may hinder clinical recognition and directly impact diagnostic strategies [7,8,9].

Historically, PCD diagnosis relied primarily on the identification of hallmark ciliary ultrastructural abnormalities by transmission electron microscopy (TEM) [10]. However, advances in molecular genetics and ciliary biology have transformed the diagnostic landscape. The recently published unified European Respiratory Society (ERS) and American Thoracic Society (ATS) guidelines (2025) proposed an evidence-based diagnostic framework that integrates clinical phenotype with complementary structural, functional, and molecular data, while recognizing that no single test is sufficient to diagnose or exclude PCD in all cases [9]. This framework emphasizes that nasal nitric oxide (nNO), high-speed video microscopy analysis (HSVA), and immunofluorescence (IF) are adjunct investigations that should be interpreted in combination with other diagnostic modalities, including transmission electron microscopy (TEM) or genetic testing, rather than as standalone diagnostic tests [9].

As part of this integrated diagnostic approach, molecular genetic testing has assumed a key role in PCD diagnosis. The identification of biallelic pathogenic or likely pathogenic variants in a known PCD-causing gene (consistent with the predominantly autosomal recessive inheritance pattern of the disease), hemizygous pathogenic variants in X-linked genes, or, more rarely, heterozygous pathogenic variants in genes with autosomal dominant inheritance, is considered definitive diagnostic evidence when consistent with the clinical phenotype [11]. To date, more than 55 genes have been implicated in PCD, accounting for approximately 65–70% of cases in well-characterized cohorts [12,13]. These genes encode proteins involved in diverse aspects of ciliary assembly, motility, and regulation, contributing to marked genetic and phenotypic heterogeneity. Consequently, next-generation sequencing (NGS), particularly whole-exome sequencing (WES) and whole-genome sequencing (WGS), has therefore emerged as a powerful diagnostic tool for PCD, enabling simultaneous evaluation of numerous disease-associated genes while requiring only minimal biological material. In addition to supporting diagnostic confirmation and elucidating disease mechanisms, genetic testing may facilitate the identification of alternative diagnoses in disorders with overlapping clinical presentations.

Despite these advances, the implementation of standardized diagnostic algorithms remains highly uneven worldwide. In low- and middle-income countries, access to specialized investigations such as nNO measurement, HSVA, TEM, and IF is frequently restricted to a small number of reference centers, contributing to under-recognition of PCD [14]. This challenge is reflected in the prolonged diagnostic odysseys experienced by many patients, often spanning years between symptom onset and etiological confirmation. In these contexts, clinical prediction tools such as the PrImary CiliAry DyskinesiA Rule (PICADAR) score are particularly valuable for identifying individuals with a high pre-test probability of PCD and prioritizing candidates for genetic testing [15].

In Brazil, studies addressing PCD diagnosis remain scarce, particularly in pediatric populations. A landmark cross-sectional study in adults with bronchiectasis demonstrated the diagnostic value of a multimodal approach combining nasal nitric oxide measurement, ciliary functional analysis, immunofluorescence, and targeted genetic testing [16]. Subsequent studies further supported the role of genetic and ultrastructural analyses in Brazilian cohorts, although access to comprehensive diagnostic tools remains limited [17]. More recently, initiatives within the Brazilian Unified Health System (SUS) have expanded access to molecular testing for rare diseases, including PCD, creating new opportunities to integrate NGS into routine clinical care [18,19,20].

In this study, we aimed to assess the clinical utility of untargeted exome sequencing (ES) in the diagnostic workup flow of Brazilian children and adolescents with suspected PCD followed at a national reference center for chronic and rare pulmonary diseases in Brazil. We describe the clinical and molecular characteristics of this pediatric cohort and evaluate the contribution of NGS to diagnostic classification in accordance with contemporary international guidelines.

## 2. Materials and Methods

### 2.1. Patients

This investigational study included children and adolescents up to 18 years and 11 months of age who were under regular follow-up at the Pediatric Pulmonology Service of the Instituto Nacional da Saúde da Criança e do Adolescente Fernandes Figueira (IFF), FIOCRUZ, Rio de Janeiro, Brazil, with a clinical suspicion of PCD. Between 2022 and 2025, all patients meeting the clinical criteria for suspected PCD were invited to participate in the study and underwent molecular investigation as part of the diagnostic evaluation and confirmation process. The ethics committee of IFF/FIOCRUZ approved the study (approval numbers 6.193.786 and 5.079.483) and all participants and their guardians signed the written informed consent.

The inclusion criteria were based on clinical characteristics of PCD as described by the ERS (European Respiratory Society), including neonatal respiratory distress, laterality defects, family history of PCD, persistent rhinorrhea, chronic rhinitis, productive cough, bronchiectasis, chronic otitis media, hearing loss, chronic rhinosinusitis and infertility. In this study, neonatal respiratory distress was defined as any respiratory difficulty in the neonatal period requiring supplemental oxygen therapy and hospitalization after birth. Clinical features were considered in an age-dependent manner.

All patients underwent a systematic evaluation for the major differential diagnoses of PCD. Individuals with alternative respiratory conditions that could mimic PCD were excluded, including those diagnosed with primary immunodeficiencies, CF, or other chronic suppurative disorders. Screening for CF included comprehensive clinical assessment and sweat chloride testing, while evaluation for immunodeficiency comprised serum immunoglobulin measurements, total lymphocyte counts, lymphocyte subset analysis, and assessment of vaccine-specific antibody responses. Chest computed tomography scans were reviewed in all cases. Assessment for chronic aspiration, when clinically indicated, included videofluoroscopic swallowing studies and bronchoscopy with bronchoalveolar lavage. After recruitment, all participants completed a clinical-epidemiological questionnaire intended to collect information on self-reported consanguinity, ethnicity, age at suspicion and the age at diagnosis, spirometry results (FEV1 and FVC), body mass index (BMI), and radiological findings were recorded. Also, the PICADAR score was applied to all cases.

Following enrollment, patients attended scheduled quarterly visits, with additional evaluations performed as clinically indicated, particularly during respiratory exacerbations or other intercurrent illnesses. At the time of manuscript submission, the median follow-up duration was 20 months (range: 3–169 months). Clinical data collected during follow-up included respiratory symptoms, nutritional status, pulmonary exacerbations, hospitalization events, microbiological findings, and pulmonary function parameters. Pulmonary function testing was performed according to the patient’s age and ability to cooperate, enabling longitudinal assessment of lung function, including forced expiratory volume in one second (FEV_1_), when available. These data were used to evaluate disease progression and long-term clinical outcomes.

### 2.2. Molecular Investigation

Before testing, all participants (or their legal guardians) received pre-test genetic counseling and provided written informed consent, including specific consent regarding the disclosure of medically actionable secondary findings. Secondary findings were managed according to current recommendations for the return of clinically actionable variants, and post-test genetic counseling was provided when such findings were identified.

To carry out this investigation, genomic DNA was isolated from the peripheral blood of the probands and, when available, their relatives using the ReliaPrep™ Blood gDNA Miniprep System (Promega Corp., Madison, WI, USA), according to the manufacturer’s instructions.

NGS was performed using either clinical exome sequencing (CES) or WES, according to the diagnostic workflow available at the time of testing. Until 2023, CES was performed using the TruSight One Expanded Sequencing Panel (Illumina Inc., San Diego, CA, USA), which targets approximately 6700 clinically relevant genes, on the Illumina NextSeq 500 (Illumina Inc., San Diego, CA, USA) platform. From 2023 onward, WES was performed using the Illumina Exome 2.0 Capture Kit (Illumina Inc., San Diego, CA, USA) on the NovaSeq 6000 (Illumina Inc., San Diego, CA, USA) or NovaSeq X Plus platforms (Illumina Inc., San Diego, CA, USA). Cases that remained unsolved after CES were subsequently evaluated by WES whenever possible, thereby increasing genomic coverage and mitigating potential differences in diagnostic performance between the two approaches.

Data processing and analysis were conducted with the Varstation platform (Varstation, Belo Horizonte, MG, Brazil) [21], employing BWA-MEM, MarkDuplicates, and IndelRealigner for read mapping; HaplotypeCaller and UnifiedGenotyper for variant calling; and Varstation Annotation for variant annotation. Variant filtering focused on single-nucleotide variants (SNVs) and small insertions/deletions located in coding and splice-site regions with an allele frequency <1%and a minimum coverage depth of >20×. A total of 193 genes were analyzed, including those 55 associated with PCD and those related to ciliopathies and differential diagnoses (Appendix A). Copy number variant (CNV) and other structural variant (SV) analyses were not included in the diagnostic workflow.

Variants were interpreted in accordance with ACMG/AMP guidelines [22], incorporating recommendations from the ClinGen Sequence Variant Interpretation (SVI) working group. Variant classification was based on multiple lines of evidence, including population frequency data (Gnomad v.4.1.1 non-UKB), published literature, disease databases, inheritance patterns, genotype–phenotype correlations, and computational predictions. For in silico evidence assessment, REVEL (≥0.644) and SpliceAI (≥0.5) were used for missense and splicing predictions, respectively, following ClinGen SVI recommendations, while AutoPVS1, DECIPHER, and InterPro supported the application of PVS1 and PM1 criteria.

Selected variants were validated by Sanger sequencing when NGS quality metrics were insufficient or for segregation analysis. Target regions containing the variants of interest were PCR-amplified using the Veriti Thermal Cycler (Thermo Fisher Scientific Inc., Waltham, MA USA) and purified with the Roche purification kit (F. Hoffmann-La Roche Ltd., Basel, Switzerland). PCR products were directly sequenced on an automated ABI 3730 DNA Analyzer (Thermo Fisher Scientific Inc., Waltham, MA, USA) with BigDye Terminator v3.1 Sequencing Buffer (Thermo Fisher Scientific Inc., Waltham, MA, USA), as described by Otto et al. [23]. The resulting chromatograms were analyzed using BioEdit software (V7.2. Ibis Biosciences, Carlsbad, CA, USA).

### 2.3. PCD Diagnosis Criteria

The classification used in the present study represents a simplified molecular adaptation of ERS/ATS Task Force. Unlike the original ERS/ATS approach, which integrates clinical, functional, ultrastructural, and genetic findings, our classification relied exclusively on molecular results obtained by WES in patients with a clinical suspicion of PCD. Individuals with biallelic variants (pathogenic or likely pathogenic) in PCD-associated genes were classified as confirmed PCD. Cases harboring a single pathogenic or likely pathogenic variant, with or without an additional variant of uncertain significance (VUS), or two variants of uncertain significance, were classified as likely PCD. This category represents a research classification created for the purposes of the present study and was not considered a definitive molecular diagnosis but rather identified individuals with genetic findings suggestive of PCD who may benefit from further investigation, including complementary functional testing or expanded molecular analysis. Individuals without relevant variants identified through genetic testing were classified as unlikely PCD.

### 2.4. Statistical Analysis

Continuous variables were summarized as medians and ranges, and categorical variables as frequencies and percentages. Comparisons of PICADAR scores across diagnostic groups were performed using the Kruskal–Wallis test. When appropriate, pairwise comparisons were conducted using the Mann–Whitney U test, with Bonferroni correction for multiple testing. Associations between categorical variables were assessed using Fisher’s exact test. A *p*-value < 0.05 was considered statistically significant.

## 3. Results

### 3.1. Demographic and Clinical Profile of Patients with Suspected Primary Ciliary Dyskinesia

**Figure 1:**
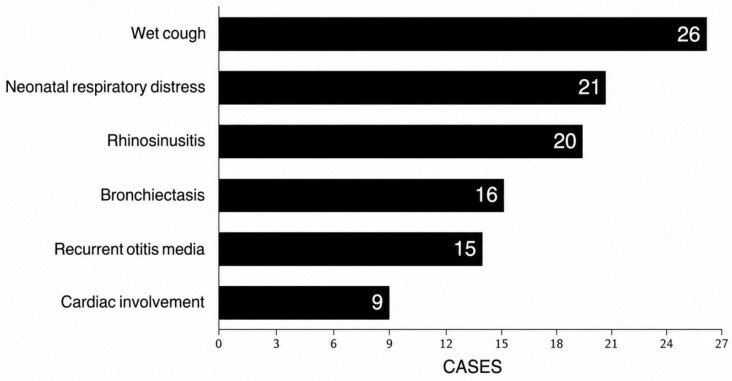
General clinical features.

A total of 27 children and adolescents with clinical suspicion of PCD were included, of whom 18 were male (67%) and 9 female (33%). The median age at first evaluation was 89 months (7.4 years; range: 1 month–18 years). No cases of parental consanguinity were reported. Neonatal respiratory distress occurred in 21 patients (77.7%), rhinosinusitis in 20 (74.1%), recurrent otitis media in 15 (55.5%), wet cough in 26 (96.3%), and cardiac involvement, predominantly laterality defects, in 9 (33.3%). High-resolution chest computed tomography revealed abnormalities in all patients, with bronchiectasis being the most frequent finding (59.2%) (Figure 1). Detailed individual clinical characteristics are provided in Table 1.

### 3.2. Molecular Spectrum of PCD-Related Variants

**Figure 2:**
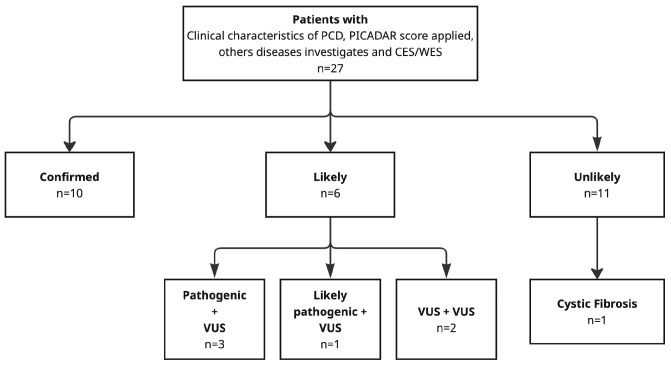
**Flowchart of patient inclusion and molecular diagnostic classification.** PCD: Primary ciliary dyskinesia. CES: Clinical-Exome Sequencing. WES: Whole-Exome Sequencing. VUS: variant of uncertain significance.

Among the 27 patients, 10 (37%) received a molecularly confirmed diagnosis of PCD established through CES/WES. Six other patients (22%) showed inconclusive genetic results and were classified as likely PCD. These included three individuals harboring one pathogenic variant and one VUS, one with a homozygous VUS, one with two VUS in compound heterozygosity, and one with one likely pathogenic variant and two VUS. In 11 cases (41%), no relevant genetic findings were identified and these individuals remain under clinical reassessment before final exclusion of a molecular diagnosis (Figure 2).

Across the cohort, 22 distinct variants were detected (Table 2), including missense (*n* = 9), nonsense (*n* = 6), frameshift (*n* = 2), splice-site (*n* = 4), and deep intronic (*n* = 1) alterations. Four variants detected in this cohort had no prior ClinVar entries. Additionally, we identified two novel variants in genes not directly associated with PCD: one in a gene of uncertain significance (GUS) and another in a gene implicated in heterotaxy, a recognized manifestation within the spectrum of PCD-associated laterality defects (Table 2).

**Figure 3:**
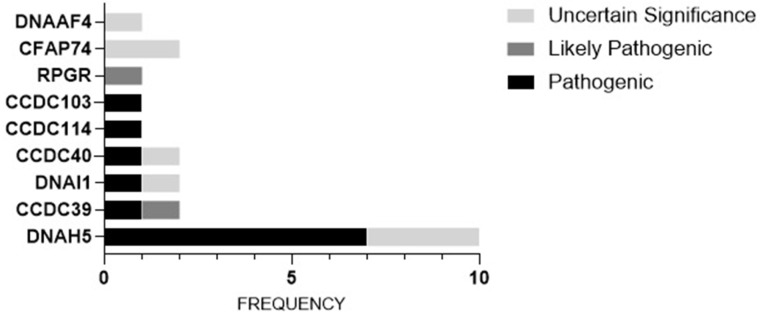
Variant Distribution Across PCD-Related Genes.

According to ACMG/AMP criteria and ClinGen SVI specifications, 11 variants (50%) were classified as pathogenic, 3 (13.6%) as likely pathogenic, and 8 (36.4%) as VUS. Variants were distributed across 9 genes, with *DNAH5* emerging as the most frequently affected. Ten distinct variants (45.4%) were identified in this gene among unrelated individuals (Figure 3).

Most VUS met one to three supporting ACMG/Clingen criteria (PM2_Supporting, PP3 or PP4). Segregation analysis of *DNAH5* c.5582A>C (Patient 1), *CCDC40* c.2597A>G (Patient 5), and *DNAI1* c.1753G>A (Patient 7), as parental DNA samples were available. However, the segregation results did not provide sufficient evidence to support reclassification of these variants as likely pathogenic. Segregation analysis was not performed for the remaining VUS due to the lack of parental DNA samples. Importantly, even if segregation had confirmed the variants to be in *trans*, the resulting application of PM3 criteria would also not have provided sufficient evidence to alter their classification (Figure 2; Table 2).

### 3.3. Notable Clinical–Genetic Findings

Case 1: Patient 13 was a female with a PICADAR score of 12 and a clinical history of chronic rhinitis and sinusitis, recurrent otitis media, persistent productive cough, recurrent pneumonia, and bronchiectasis. Imaging studies revealed *situs inversus totalis* with dextrocardia. Genetic analysis identified two heterozygous variants in *CCDC39*, one pathogenic (c.1167+1261A>G) and one likely pathogenic (c.210+2T>C), both previously associated with PCD, as well as a third likely pathogenic variant (c.195dup; p.Glu66ArgfsTer52) in *CFC1* gene, related to Heterotaxy.

Case 2: Patient 4 was a male who developed respiratory symptoms at 94 months of age, with a PICADAR score of 2. He presented with short stature, chronic rhinitis and sinusitis, chronic wet cough, but had no history of neonatal respiratory distress, recurrent otitis media, or ocular findings. Chest computed tomography demonstrated bronchiectasis, and cardiac situs was normal. Genetic testing identified a hemizygous likely pathogenic variant in *RPGR* (c.2072C>A; p.Ser691*) related to Retinopathy and sinorespiratory infections, along with an incidental heterozygous likely pathogenic variant in *FGFR3* (c.1620C>A; p.Asn540Lys) (*FGFR3*-related Hypochondroplasia).

Case 3: Patient 14 was a 15-year-old female initially suspected of having PCD, with a PICADAR score of 7. Sweat chloride test (17 mmol/L), immunological evaluation and fecal elastase levels were normal. Chest imaging demonstrated bilateral bronchiectasis consistent with chronic suppurative lung disease, while microbiological analysis was positive only for *Staphylococcus aureus.* Genetic testing revealed two previously reported heterozygous pathogenic variants in *CFTR*: c.1521_1523del (p.Phe508del) and c.3454G>C (p.Asp1152His), a genotype previously associated with CF. Clinical follow-up demonstrated progressive pulmonary disease and subsequent clinical and functional improvement after CFTR modulator therapy.

Case 4: Patient 22 was a female who developed respiratory symptoms at 116 months of age, with a PICADAR score of 6. She had a history of neonatal respiratory distress, chronic rhinitis, and sinusitis, but no history of chronic wet cough or recurrent otitis media. Imaging studies demonstrated *situs inversus totalis* and bronchiectasis. No pathogenic or likely pathogenic variants were identified in known PCD-associated genes. However, genetic analysis revealed a heterozygous frameshift variant in *DNAH14* (c.102dup; p.Tyr35IlefsTer2), which was classified as a VUS, as *DNAH14* is currently considered a gene of uncertain relevance to PCD. Accordingly, this finding was not considered diagnostic, and the patient remained classified as unlikely PCD. In addition, a heterozygous pathogenic variant was identified in *CFTR* (c.3154T>G; p.Phe1052Val).

### 3.4. Molecular Diagnostic Classification and PICADAR Score

**Figure 4:**
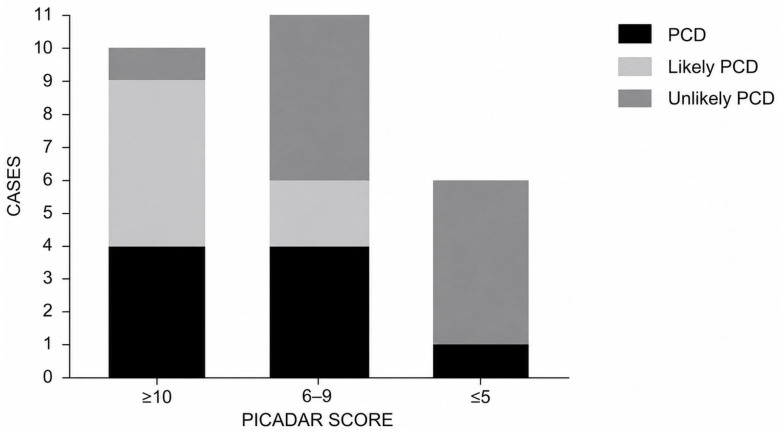
Distribution of Molecular Diagnostic Outcomes According to PICADAR Scores.

Clinical characteristics varied across the three diagnostic outcome groups (confirmed PCD, likely PCD and unlikely) and are summarized in Table 3 and Figure 4. Patients with confirmed PCD exhibited the highest burden of hallmark features, including neonatal respiratory distress (80%), chronic wet cough (100%), chronic rhinitis (80%), and laterality defects (40%), with a median PICADAR score of 8 (range: 6–14). Individuals in the likely PCD group exhibited several shared features with confirmed cases, particularly upper airway disease and bronchiectasis, and showed a slightly higher median PICADAR score of 10 (range: 3–10). In contrast, patients in the unlikely group displayed fewer classic indicators, including lower frequencies of neonatal distress and laterality defects, and had a lower median PICADAR score of 6 (range: 2–10).

PICADAR scores were compared across the three diagnostic groups using the Kruskal–Wallis test, and no statistically significant differences were observed (*p* = 0.059). Among individuals with PICADAR scores ≥ 10 (*n* = 10), four cases had a positive molecular diagnosis, five were classified as likely PCD (Figure 4) and one case had a negative result. In the intermediate PICADAR group (scores 6–9; *n* = 11), four individuals presented a positive molecular diagnosis, two were classified as likely PCD, and five had negative results. In contrast, among individuals with low PICADAR scores (≤5; *n* = 6), molecular diagnostic outcomes were predominantly negative, with five negative cases and only one positive diagnosis; no cases were classified as likely PCD in this group.

Analysis of PICADAR as a quantitative variable suggested higher scores in the likely PCD groups compared to the unlikely group (*p* = 0.029). However, no pairwise comparison remained statistically significant after correction for multiple testing.

## 4. Discussion

This study provides a molecular characterization of a pediatric cohort with suspected PCD in Brazil, a setting where access to comprehensive diagnostic approaches remains limited. Among the 27 children and adolescents evaluated, 10 received a confirmed genetic diagnosis, and an additional 6 were classified as likely PCD based on combined clinical and molecular evidence (Figure 2), highlighting the importance of integrating genetic findings with phenotypic assessment in this heterogeneous condition [4,24]. Furthermore, WES enabled the identification of additional clinically relevant findings, including one case of CF, one diagnosis of *FGFR3*-related hypochondroplasia, and one secondary finding involving a pathogenic *BRCA2* variant.

Using exome sequencing as the primary diagnostic strategy, we achieved a molecular diagnostic yield of 37%. Recent studies have demonstrated the value of early incorporation of NGS into the diagnostic evaluation of selected PCD cohorts [25,26,27,28,29,30,31]. Reported molecular diagnosis rates in PCD vary widely, ranging from approximately 17% to over 94% [25,26,27,28,29,32,33,34,35], reflecting differences in clinical selection criteria, population characteristics, and genetic testing methodologies. Higher yields are generally observed in phenotypically well-characterized populations and in cohorts with higher rates of consanguinity [25,27,36,37,38,39,40]. In our cohort, the absence of systematic implementation of the multimodal ERS/ATS diagnostic algorithm may have contributed to diagnostic uncertainty and a lower overall molecular diagnostic yield [11,16,41]. Nevertheless, our findings support the value of ES as an early diagnostic tool for suspected PCD.

The marked genetic heterogeneity of PCD continues to shape diagnostic outcomes, with the ongoing discovery of disease-associated genes progressively improving molecular diagnosis [13,42]. However, a considerable proportion of clinically well-characterized patients still lack a definitive molecular diagnosis, with approximately one in four to one in five cases remaining genetically unresolved [4,11,27]. In this study, CES was initially applied in a sequential workflow, followed by a transition to exclusive use of untargeted WES from 2023 onward. This shift reflects the broader genomic scope of WES, which enables the detection of variants in both established and newly described PCD-associated genes, thereby increasing the likelihood of identifying rare or unexpected genetic causes compared to more restricted panels [43]. In addition, WES allowed the identification of alternative diagnoses and secondary findings, which were not assessed with CES (Table 2).

Despite these advantages, negative molecular findings may still reflect inherent limitations of WES. As this approach primarily targets coding and canonical splice regions, pathogenic variants located in deep intronic or regulatory regions may remain undetected. In addition, WES has limited sensitivity for copy number variants (CNV), an important contributor to PCD, including recurrent *DNAH5* deletions, and faces technical challenges in the analysis of genes with highly homologous regions, such as *HYDIN*, due to mapping ambiguity [44,45,46]. Although expanded capture designs may occasionally identify non-canonical variants, as illustrated by the detection of a deep intronic *CCDC39* variant in our cohort, these remain exceptions [47,48,49]. Therefore, some unresolved cases in our cohort, particularly those with high PICADAR scores, may harbor undetected CNVs or other genomic alterations not readily detectable by WES. Consistent with this, Black et al. [29] demonstrated that WGS can improve the molecular diagnosis of PCD by identifying structural and other pathogenic variants missed by exome-based approaches. Together, these findings highlight the potential value of complementary genomic strategies, including WGS, long-read technologies, transcript-level analyses, and targeted CNV detection, to improve diagnostic completeness.

Variant interpretation represents an additional challenge to diagnostic yield, as VUS are common and the way they are classified and integrated with clinical findings can substantially influence whether a case is ultimately considered diagnostic or non-diagnostic [11]. Across studies, the classification of cases harboring VUS has not been uniform, reflecting differences in methodological rigor and the availability of complementary diagnostic data. In more conservative approaches, patients with only VUS are typically classified as inconclusive or non-diagnostic [17,28]. In contrast, other studies have incorporated VUS into a positive or likely diagnosis when supported by strong clinical, functional, or structural evidence, such as characteristic defects on transmission electron microscopy or abnormal nasal nitric oxide levels [11,16,50]. In accordance with current ERS/ATS diagnostic terminology, the presence of one or more VUS alone does not establish a definitive diagnosis of PCD [9]. In our cohort, individuals harboring VUS were classified as likely PCD, a category that does not represent a definitive diagnosis but rather identifies patients who should be considered candidates for additional molecular analysis.

VUS were a major contributor to the moderate diagnostic yield. Eight variants were classified as VUS, most of which were rare missense changes with uncertain biological impact and supported only by limited ACMG/ClinGen evidence. The lack of functional validation, segregation data, and robust population frequency information impaired variant interpretation, contributing to inconclusive diagnostic [11,24]. Notably, re-evaluation under updated ACMG/AMP [22] and ClinGen SVI recommendations [51] led to the reclassification of variants and a reduction in molecular diagnostic yield from a potential 60% to 37%, underscoring the impact of evolving interpretation frameworks. Given the dynamic nature of VUS classification, periodic reanalysis and the integration of complementary approaches are essential to improve diagnostic resolution over time.

Consistent with previous Brazilian and international studies, *DNAH5* was the most frequently affected gene in our cohort (43.4%), reinforcing its central role in PCD pathogenesis [24,52]. Most variants were classified as pathogenic or likely pathogenic (*n* = 7), with only three classified as VUS. One VUS, *DNAH5* c.5582A>C (p.Gln1861Pro), would require at least one additional moderate ACMG criterion for reclassification as likely pathogenic. The remaining VUS (*DNAH5* c.12037 C>T; p.Arg4013Cys and c.10657 C>T; p.Arg3553Trp) were supported only by supporting-level evidence (PM2_Supporting and PP4) in one individual with high PICADAR scores, underscoring the need for complementary diagnostic approaches and continued evidence accumulation.

The identification of novel and rare variants, expands the mutational spectrum of PCD and reflects the marked genetic heterogeneity of the disease [9]. Variant distribution differs substantially across populations and is often shaped by regional founder effects rather than a single recurrent mutation worldwide. Distinct founder variants have been described in specific populations, contributing to local enrichment of certain genotypes [53,54,55,56,57]. In contrast, the highly admixed genetic background of the Brazilian population likely results in a more diverse and less recurrent mutational profile [16,17]. This genetic diversity may partially contribute to the high proportion of VUS observed in our cohort, reflecting the limited availability of population-specific data and the challenges of variant interpretation in underrepresented populations.

Beyond the primary diagnosis, WES provided additional clinical value by enabling the identification of secondary and incidental findings, allowing the recognition of overlapping or unrelated conditions with direct impact on clinical management and genetic counseling. This expanded diagnostic scope highlights its ability to reveal clinically subtle or previously unrecognized syndromic conditions, as well as actionable findings outside the initial diagnostic hypothesis. For example, in Patient 4 the incidental identification of a pathogenic *FGFR3* variant led to the recognition of previously unsuspected skeletal abnormalities consistent with hypochondroplasia, while a pathogenic *BRCA2* variant identified in Patient 8 had implications for long-term surveillance and genetic counseling. In Patient 14, WES enabled the diagnosis of a CF, refining the differential diagnosis in a case with overlapping respiratory features, as this condition can mimic PCD but carries distinct therapeutic and prognostic implications [58]. In this case, the combination of F508del and D1152H variants accounted for the normal sweat chloride levels and the presence of clinically significant pulmonary disease, characterized by bilateral bronchiectasis and progressive decline in lung function. The D1152H variant is a Class IV CFTR mutation associated with residual CFTR function, which may explain the absence of significant gastrointestinal manifestations and normal fecal elastase levels. In contrast, F508del, the most common CF-causing variant, results in defective CFTR protein processing and severe loss of channel function. Consistent with previous reports, the combination of a severe CF-causing allele and a residual-function variant, such as F508del/D1152H, is associated with considerable phenotypic variability. While residual CFTR function may attenuate extrapulmonary manifestations, it does not necessarily prevent the development of progressive chronic lung disease, as observed in this case [59,60,61,62]. Importantly, this finding was clinically actionable, allowing the implementation of CFTR modulator therapy that improved the patient’s quality of life, highlighting the discriminatory power of WES. Additional complexity was observed in Patient 13, who presented with classical PCD features and *situs inversus totalis*, and harbored biallelic variants in *CCDC39*, that fully explain the PCD phenotype. However, the presence of a concomitant variant in *CFC1*, a gene involved in left-right axis determination during embryogenesis, introduced interpretative challenges, as both genes may contribute to overlapping features such as laterality defects [63,64]. Furthermore, in Patient 22, no pathogenic variants were identified in established PCD-associated genes despite presenting hallmark features of PCD, including *situs inversus totalis* and bronchiectasis. Instead, WES detected a rare heterozygous frameshift variant in *DNAH14*, a gene not routinely included in clinical PCD panels due to its uncertain disease association and currently designated as having disputed evidence by Motile Ciliopathy Gene Curation Expert Panel (CCID:009010). Although current evidence linking *DNAH14* to PCD remains limited [50], its identification underscores the limitations of restricted gene panels and reinforces the value of comprehensive exome-wide analysis in genetically heterogeneous disorders. As the current molecular findings do not establish a definitive diagnosis, the patient remains under clinical follow-up and is currently undergoing WGS to further investigate the underlying genetic cause.

Diagnostic yield was associated with clinical presentation and PICADAR score (Table 3; Figure 4). Classical PCD features, including neonatal respiratory distress, situs abnormality and congenital heart defect, remain strong clinical predictors and are key components of the PICADAR score [65]. In our cohort, higher PICADAR scores (≥6) were more frequently observed among individuals with confirmed or likely molecular diagnoses, whereas lower scores (≤5) predominated in negative cases, with diagnostic yields of 50%, 36%, and 16% across the high (≥10), intermediate (6–9), and low-score (≤5) groups, respectively. However, differences in PICADAR scores between diagnostic groups did not remain statistically significant after correction for multiple testing, possibly reflecting the small sample size and consequent reduction in statistical power. Nevertheless, the observed distribution of scores is consistent with the role of PICADAR as a screening and prioritization tool for genetic testing, particularly in resource-limited settings, in agreement with ERS/ATS recommendations that recognize symptom-based tools as practical and cost-effective approaches for identifying individuals with a high likelihood of PCD [9].

While these findings support the usefulness of PICADAR as a screening and prioritization tool, they also highlight the limitations of phenotype-based scoring systems in the context of the marked clinical heterogeneity of PCD, particularly in individuals with atypical presentations and without laterality defects. As the only individual with a confirmed molecular diagnosis in the low PICADAR group, this patient presented relevant respiratory features but lacked several classical criteria, underscoring the heterogeneous presentation of PCD and the potential for affected individuals to be underrecognized by phenotype-based prediction tools. Additionally, a likely pathogenic variant in *RPGR* was identified in this patient. Although this gene is classically associated with X-linked retinitis pigmentosa, growing evidence supports its role in motile cilia dysfunction and PCD, including reports of individuals presenting predominantly with respiratory manifestations [16,66,67]. The absence of ocular manifestations in our patient further expands the phenotypic spectrum associated with *RPGR*. The relatively mild respiratory phenotype observed highlights the variable expressivity and incomplete penetrance of *RPGR*-associated disease, as also illustrated by a similar Brazilian case reported by Olm et al. [16]. The patient was referred for ophthalmological evaluation to investigate possible subclinical ocular involvement associated with this gene; however, follow-up data are not yet available.

Overall, these findings demonstrate that combining structured clinical assessment, imaging, and comprehensive molecular testing (ideally as early as possible) can substantially improve diagnostic accuracy and reduce delays in care. At the time of this study, ERS and ATS recommendations already supported genetic testing in selected PCD cases, and the recent ERS/ATS guideline (2025) further reinforces NGS as a key component of the diagnostic pathway, particularly where specialized functional tests are not available. Although molecular confirmation was achieved in only a subset of patients, these findings support the value of genetic testing as part of a comprehensive diagnostic approach, while also illustrating that WES cannot yet resolve all clinically suspected cases because of both technical limitations and the incomplete understanding of the genetic basis of PCD. In Brazil, where diagnostic tools such as TEM and nasal nitric oxide remain restricted, NGS represents a scalable diagnostic approach aligned with national precision medicine initiatives, including Brazilian health policies that have supported the incorporation of ES into the diagnostic evaluation of rare diseases within the public health system [18,68,69]. Broader implementation of NGS-based evaluation has potential to reduce the diagnostic odyssey, facilitate access to appropriate clinical management, and support genetic counseling and reproductive decision-making.

## 5. Conclusions

In conclusion, this study expands the molecular landscape of PCD in a Brazilian pediatric population and demonstrates the clinical utility of ES in low- and middle-income countries. Beyond confirming PCD diagnoses, WES enabled the identification of alternative genetic conditions with overlapping phenotypes, improving diagnostic accuracy and directly influencing patient management, while underscoring the importance of multidisciplinary evaluation when complex or incidental findings are identified. Our findings also support the value of PICADAR as a practical tool for patient stratification and prioritization of genetic testing. Nevertheless, the lack of complementary ERS/ATS-recommended diagnostic tests and the inability of WES to detect all classes of genomic variation remain important barriers to achieving a definitive diagnosis in every case. Taken together, our findings support the integration of genomic testing into the diagnostic workup of suspected PCD while underscoring the need to expand access to comprehensive diagnostics for rare pulmonary diseases.

## Figures and Tables

**Table 1:** Clinical data and summary for the 27 patients. NRD—Neonatal respiratory distress; VSD—Ventricular Septal Defect; ASD—Atrial Septal Defect; CES: clinical-exome sequencing. WES: Whole-exome sequencing. SIT: *Situs Inversus Totalis*. LFH: Left ventricular hypoplasia. DX: Dextrocardia.

Patient ID	Molecular Method	Gender	Age at First Assessment (Months)	Age at Diagnosis (Months)	PICADAR	NRD	Wet Cough	Rhino Sinusitis	Otitis Media	Cardiac Involvement	Bronchiectasis	Final Molecular Diagnosis
1	CES	Male	95	188	10	Yes	Yes	Yes	Yes	SIT	Yes	Likely
2	CES	Male	126	186	8	Yes	Yes	Yes	No	-	Yes	Confirmed
3	CES	Male	120	244	8	Yes	Yes	Yes	Yes	-	Yes	Confirmed
4	WES	Male	94	246	2	No	Yes	Yes	No	-	Yes	Confirmed
5	CES	Male	44	160	11	Yes	Yes	Yes	No	DX	Yes	Likely
6	WES	Male	37	173	7	Yes	Yes	Yes	Yes	-	Yes	Unlikely
7	CES	Male	1	103	14	Yes	Yes	Yes	Yes	SIT, ASD, PDA	Yes	Likely
8 ^1^	WES	Male	42	47	5	Yes	Yes	Yes	No	-	No	Unlikely
9 ^1^	WES	Male	18	31	4	Yes	Yes	Yes	Yes	-	No	Unlikely
10	WES	Female	56	80	4	No	Yes	No	Yes	-	Yes	Unlikely
11	WES	Male	193	209	8	Yes	Yes	Yes	Yes	-	Yes	Unlikely
12	WES	Female	119	138	8	Yes	Yes	Yes	Yes	-	No	Unlikely
13	WES	Female	109	130	12	Yes	Yes	Yes	Yes	SIT	Yes	Confirmed
14	WES	Female	128	187	7	Yes	Yes	Yes	No	-	Yes	Unlikely
15	WES	Female	138	219	5	No	Yes	No	Yes	-	Yes	Unlikely
16	WES	Male	126	127	7	Yes	Yes	Yes	Yes	-	Yes	Confirmed
17 ^2^	WES	Male	89	90	6	Yes	Yes	Yes	Yes	-	No	Confirmed
18 ^2^	WES	Male	67	69	10	Yes	Yes	Yes	Yes	LFH	Yes	Confirmed
19 ^3^	WES	Male	112	118	12	Yes	Yes	Yes	Yes	DX, SIT	Yes	Confirmed
20 ^3^	WES	Female	11	18	6	No	Yes	Yes	No	DX, SIT	No	Confirmed
21	WES	Male	1	14	10	Yes	No	No	No	DX, SIT	No	Confirmed
22	WES	Female	116	128	6	Yes	Yes	Yes	No	SIT	No	Unlikely
23	WES	Female	31	101	10	Yes	Yes	No	No	SIT	Yes	Unlikely
24	WES	Male	217	232	3	No	Yes	No	No	-	No	Unlikely
25	WES	Female	21	36	6	Yes	Yes	No	No	-	No	Likely
26	WES	Male	8	21	10	Yes	Yes	No	No	SIT	No	Likely
27	WES	Male	35	36	10	No	Yes	Yes	Yes	SIT, VSD, ASD	No	Likely

^1,2,3^ Siblings.

**Table 2:** Molecular findings and variant classification in Brazilian paediatric patients with suspected primary ciliary dyskinesia. PCD: Primary Ciliary Dyskinesia associated; SF: Secondary finding; AD: Alternative diagnosis; GUS: Gene of Uncertain Significance; IF: Incidental Finding. Comp Het: Compound Heterozygous. Het: Heterozygous: Hom: Homozygous; VUS: Variant of Uncertain Significance. P: Pathogenic. LP: Likely Pathogenic. DR: Drug response.

Patient ID	Gene	Transcript	cDNA	Protein	Clinvar Accession	Zygosity	Segregation Analysis	Impact	ACMG Criteria ^1^	ACMG Classification	Clinical Relevance
1	*DNAH5*	NM_001369.2	c.5582A>C	p.Gln1861Pro	VCV000977555.8	Comp Het	Paternal	Missense	PM2_P, PM3, PP4	VUS	PCD
	*DNAH5*	NM_001369.2	c.11653C>T	p.Arg3885Ter	VCV000525218.22	Comp Het	Maternal	Nonsense	PVS1, PM2_P, PM3, PP4	P	
2	*DNAH5*	NM_001369.2	c.2283_2284del	p.Arg761SerfsTer10	VCV000523616.19	Comp Het	Paternal	Frameshift	PVS1, PM2_P, PM3_S, PP1_M, PP4	P	PCD
	*DNAH5*	NM_001369.2	c.11653C>T	p.Arg3885Ter	VCV000525218.22	Comp Het	Maternal	Nonsense	PVS1, PM2_P, PP4	P	
3	*CCDC114*	NM_001364171.2	c.59+1G>A	-	VCV001799681.2	Hom	Biparental	Splice donor	PVS1, PM2_P, PM3_S, PP4	P	PCD
4	*RPGR*	NM_000328.3	c.2072 C>A	p.Ser691Ter	Novel	Hem	Unknown	Nonsense	PVS1, PM2_P	LP	PCD
	*FGFR3*	NM_000142.5	c.667C>T	p.Arg223Cys	VCV000838879.25	Het	Unknown	Missense	PS4_M, PM2_P, PP1_M, PP3	LP	IF
5	*CCDC40*	NM_017950.4	c.961C>T	p.Arg321Ter	VCV000216118.16	Comp Het	Paternal	Nonsense	PVS1, PM2_P, PP4	P	PCD
	*CCDC40*	NM_017950.4	c.2597A>G	p.Asn866Ser	VCV000861549.10	Comp Het	Maternal	Missense	PM2_P, PM3, PP4	VUS	
7	*DNAI1*	NM_012144.4	c.1543G>A	p.Gly515Ser	VCV000005606.20	Comp Het	Paternal	Missense	PM2_P, PM3_S, PP1_M, PP3, PP4	P	PCD
	*DNAI1*	NM_012144.4	c.1753G>A	p.Val585Met	VCV003503796.1	Comp Het	Maternal	Missense	PM2_P, PM3, PP3, PP4	VUS	
8	*BRCA2*	NM_001432077.1	c.8488-1G>A	-	VCV000038164.89	Het	Maternal	Splicing acceptor	PVS1, PM2_P, PS1_Sup	P	SF
13	*CCDC39*	NM_181426.2	c.210+2T>C	-	VCV001916247.7	Comp Het	Unknown	Splicing donor	PVS1_M, PM2_P, PM3, PP4	LP	PCD
	*CCDC39*	NM_181426.2	c.1167+1261A>G	p.Glu390SerfsTer6	VCV000410378.17	Comp Het	Unknown	Deep Intronic	PVS1, PM3, PM2_P, PP4	P	
	*CFC1*	NM_032545.4	c.195dup	p.Glu66ArgfsTer52	Novel	Het	Unknown	Frameshift	PVS1, PM2_P	LP	AD
14	*CFTR*	NM_000492.4	c.1521_1523del	p.Phe508del	VCV000007105.206	Comp Het	Unknown	Frameshift	PS3_P, PM1, PM3, PM4	P	AD
	*CFTR*	NM_000492.4	c.3454G>C	p.Asp1152His	VCV000035867.102	Comp Het	Unknown	Missense	PS1, PS3_P, PM1, PM3	P/DR	
16	*DNAH5*	NM_001369.2	c.11536C>T	p.Gln3846Ter	VCV000944438.10	Comp Het	Unknown	Nonsense	PVS1, PM2_P, PM3_P	P	PCD
	*DNAH5*	NM_001369.2	c.4237C>T	p.Gln1413Ter	VCV001458964.8	Comp Het	Unknown	Nonsense	PVS1, PM2_P, PM3_S	P	
17	*DNAH5*	NM_001369.2	c.2283_2284del	p.Arg761SerfsTer10	VCV000523616.19	Comp Het	Unknown	Frameshift	PVS1, PM2_P, PM3_S, PP1_M	P	PCD
	*DNAH5*	NM_001369.2	c.10555+1G>A	-	Novel	Comp Het	Unknown	Splicing donor	PVS1, PS1_P, PM2_P, PM3, PP1_M	P	
18	*DNAH5*	NM_001369.2	c.2283_2284del	p.Arg761SerfsTer10	VCV000523616.19	Comp Het	Unknown	Frameshift	PVS1, PM2_P, PM3_S, PP1_M	P	PCD
	*DNAH5*	NM_001369.2	c.10555+1G>A	-	Novel	Comp Het	Unknown	Splicing donor	PVS1, PS1_P, PM2_P, PM3, PP1_M	P	
19	*DNAH5*	NM_001369.2	c.13194_13197del	p.Asp4398GlufsTer16	VCV000163134.47	Hom	Unknown	Frameshift	PVS1, PM2_P, PP1_M, PP4	P	PCD
20	*DNAH5*	NM_001369.2	c.13194_13197del	p.Asp4398GlufsTer16	VCV000163134.47	Hom	Unknown	Frameshift	PVS1, PM2_P, PP1_M, PP4	P	PCD
21	*CCDC103*	NM_213607.3	c.461A>C	p.His154Pro	VCV000031698.83	Hom	Unknown	Missense	PP1_S, PS3_P, PM2_P, PM3	LP	PCD
22	*CFTR*	NM_000492.4	c.3154T>G	p.Phe1052Val	VCV000035865.107	Het	Unknown	Missense	PM1, PM2_P, PP3, PM3	LP/DR	AD
	*DNAH14*	NM_001367479.1	c.102dup	p.Tyr35IlefsTer2	Novel	Het	Unknown	Frameshift	Not Applied	VUS	GUS
25	*CFAP74*	NM_001304360.2	c.1222G>T	p.Val408Phe	Novel	Comp Het	Unknown	Missense	PM2_P	VUS	PCD
	*CFAP74*	NM_001304360.2	c.4279G>A	p.Val1427Ile	Novel	Comp Het	Unknown	Missense	PM2_P	VUS	
26	*DNAAF4*	NM_130810.4	c.406-10A>G	-	VCV000454961.10	Hom	Unknown	Splicing	PM2_P, PP4	VUS	PCD
27	*DNAH5*	NM_001369.2	c.12397 G>T	p.Glu4133Ter	VCV001344889.9	Comp Het	Unknown	Nonsense	PVS1, PM2_P, PM3_P, PP4	LP	PCD
	*DNAH5*	NM_001369.2	c.12037 C>T	p.Arg4013Cys	VCV002140315.5	Comp Het	Unknown	Missense	PM2_P, PP4	VUS	
27	*DNAH5*	NM_001369.2	c.10657 C>T	p.Arg3553Trp	VCV001583038.8	Comp Het	Unknown	Missense	PM2_P, PP4	VUS	PCD

^1^ Criteria applied according to ACMG/AMP and ClinGen SVI recommendations. ACMG evidence codes are annotated with strength modifiers in the table (e.g., PS4_M indicates PS4 with moderate strength; PS4_S indicates strong strength; PS4_P indicates supporting strength).

**Table 3:** Clinical characteristics of patients according to molecular diagnostic classification for primary ciliary dyskinesia. PCD: primary ciliary dyskinesia; yr: years; m: months.

General Clinical Features	Group		
	Confirmed PCD (*n* = 10)	Likely PCD (*n* = 6)	Unlikely PCD (*n* = 11)
Gender (male)	8 (80%)	5 (83%)	5 (45%)
Age at assessment-Median (range), yr ^1^	8.4 (1 m–10.5 yr)	2.7 (1 m–18 yr)	8 (1.5 yr–16.1 yr)
PICADAR score (median)	8 (6–14)	10 (3–10)	6 (2–10)
Neonatal respiratory distress	8 (80%)	5 (83%)	8 (73%)
Chronic cough	10 (100%)	5 (83%)	11 (100%)
Chronic rhinitis	8 (80%)	4 (66%)	9 (82%)
Situs inversus totalis	4 (40%)	4 (66%)	2 (18%)
Heterotaxis or congenital heart disease	6 (60%)	5 (83%)	2 (18%)
Recurrent otitis	6 (60%)	3 (50%)	6 (54,5%)
Bronchiectasis	6 (60%)	4 (66%)	5 (45%)

^1^ Age was adjusted in the model.

## Data Availability

The original contributions presented in this study are included in the article. Further inquiries can be directed to the corresponding authors.

## Abbreviations

The following abbreviations are used in this manuscript:

PCD	Primary Ciliary Dyskinesia
VUS	Variant of Uncertain Significance
GUS	Gene of Uncertain Significance
NGS	Next-generation sequencing
WES	Whole Exome Sequencing

## Appendix A

Variant analysis was initially restricted to genes previously associated with initial clinical suspicion (PCD) and differential diagnosis: *ACBD3*, *ACVR2B*, *ADGRV1*, *AGPAT2*, *AHI1*, *AIPL1*, *AK7*, *AKNA*, *ARL13B*, *ARL6*, *ARMC2*, *B9D1*, *B9D2*, *BBS1*, *BBS10*, *BBS12*, *BBS2*, *BBS4*, *BBS5*, *BBS7*, *BBS9*, *BRWD1*, *CC2D2A*, *CCDC103*, *CCDC28B*, *CCDC39*, *CCDC40*, *CCDC50*, *CCDC65*, *CCDC8*, *CCNO*, *CDH23*, *CDKN1C*, *CENPF*, *CEP164*, *CEP290*, *CEP41*, *CEP55*, *CFAP20DC*, *CFAP221*, *CFAP298*, *CFAP300*, *CFAP43*, *CFAP46*, *CFAP47*, *CFAP53*, *CFAP54*, *CFAP57*, *CFAP74*, *CFC1*, *CFTR*, *CLRN1*, *CLXN*, *CRB1*, *CRELD1*, *CRX*, *DAW1*, *DNAAF1*, *DNAAF11*, *DNAAF2*, *DNAAF3*, *DNAAF4*, *DNAAF5*, *DNAAF6*, *DNAH1*, *DNAH10*, *DNAH11*, *DNAH12*, *DNAH14*, *DNAH17*, *DNAH5*, *DNAH6*, *DNAH7*, *DNAH8*, *DNAH9*, *DNAI1*, *DNAI2*, *DNAJB13*, *DNAL1*, *DRC1*, *DYNC2H1*, *ENKUR*, *EVC*, *EVC2*, *FOXH1*, *FOXJ1*, *GAS2L2*, *GAS8*, *GDF1*, *GLIS2*, *GOLGA3*, *GUCY2D*, *HYDIN*, *HYLS1*, *IFT43*, *IFT74*, *IFT80*, *IMPDH1*, *INVS*, *IQCB1*, *ITCH*, *KCNJ13*, *KCNN3*, *KIF7*, *LCA5*, *LEFTY2*, *LRAT*, *LRRC56*, *LZTFL1*, *MCIDAS*, *MCM4*, *MKKS*, *MKS1*, *MNS1*, *MYO7A*, *NEK1*, *NEK10*, *NEK8*, *NFKB1*, *NFKB2*, *NKX2-5*, *NME1*, *NME5*, *NME8*, *NODAL*, *NPHP1*, *NPHP3*, *NPHP4*, *ODAD1*, *ODAD2*, *ODAD3*, *ODAD4*, *OFD1*, *PCDH15*, *PIK3CD*, *PIK3R1*, *PKD2*, *PKHD1*, *RAG1*, *RAG2*, *RD3*, *RDH12*, *RPE65*, *RPGR*, *RPGRIP1*, *RPGRIP1L*, *RSPH1*, *RSPH3*, *RSPH4A*, *RSPH9*, *SCNN1A*, *SCNN1B*, *SCNN1G*, *SDCCAG8*, *SPAG1*, *SPAG16*, *SPAG17*, *SPATA7*, *SPEF2*, *STK36*, *TCTN1*, *TCTN2*, *TEKT1*, *TMEM138*, *TMEM216*, *TMEM231*, *TMEM237*, *TMEM67*, *TOPORS*, *TP73*, *TRIM32*, *TSC1*, *TSC2*, *TTC12*, *TTC21B*, *TTC8*, *TTLL6*, *TUBB4B*, *TULP1*, *UMOD*, *USH1C*, *USH1G*, *USH2A*, *VHL*, *WDPCP*, *WDR19*, *WDR35*, *WHRN*, *XPNPEP3*, *ZAP70*, *ZIC3*, *ZMYND10*, *ZNF423*. The filtering of variants was directed towards coding or splicing regions, with allelic frequency <1% (gnomAD and ABraOM) and coverage depth >20x.
